# Advanced Fault Diagnosis Methods in Molecular Networks

**DOI:** 10.1371/journal.pone.0108830

**Published:** 2014-10-07

**Authors:** Iman Habibi, Effat S. Emamian, Ali Abdi

**Affiliations:** 1 Electrical and Computer Engineering Department, New Jersey Institute of Technology, Newark, New Jersey, United States of America; 2 Advanced Technologies for Novel Therapeutics (ATNT), Enterprise Development Center, Newark, New Jersey, United States of America; 3 Biological Sciences Department, New Jersey Institute of Technology, Newark, New Jersey, United States of America; Tel Aviv University, Israel

## Abstract

Analysis of the failure of cell signaling networks is an important topic in systems biology and has applications in target discovery and drug development. In this paper, some advanced methods for fault diagnosis in signaling networks are developed and then applied to a caspase network and an SHP2 network. The goal is to understand how, and to what extent, the dysfunction of molecules in a network contributes to the failure of the entire network. Network dysfunction (failure) is defined as failure to produce the expected outputs in response to the input signals. Vulnerability level of a molecule is defined as the probability of the network failure, when the molecule is dysfunctional. In this study, a method to calculate the vulnerability level of single molecules for different combinations of input signals is developed. Furthermore, a more complex yet biologically meaningful method for calculating the multi-fault vulnerability levels is suggested, in which two or more molecules are simultaneously dysfunctional. Finally, a method is developed for fault diagnosis of networks based on a ternary logic model, which considers three activity levels for a molecule instead of the previously published binary logic model, and provides equations for the vulnerabilities of molecules in a ternary framework. Multi-fault analysis shows that the pairs of molecules with high vulnerability typically include a highly vulnerable molecule identified by the single fault analysis. The ternary fault analysis for the caspase network shows that predictions obtained using the more complex ternary model are about the same as the predictions of the simpler binary approach. This study suggests that by increasing the number of activity levels the complexity of the model grows; however, the predictive power of the ternary model does not appear to be increased proportionally.

## Introduction

Analysis of molecular networks using a variety of engineering and computational tools and approaches has been an active area of research in systems biology in recent years [1]. Molecular systems biology looks at the orchestrated function of the molecular components and their complex interactions within the cell, and typically involves studies on metabolic networks or cell signaling networks, using a holistic approach to molecular biology research [1]. Systems biology makes heavy use of mathematical and computational models to understand the pathology of networks, to develop methods to quantify the functions of molecules within a network, to eventually understand their roles in the possible malfunction of the network. Molecular fault diagnosis engineering was introduced in recent years [2] [3], to find the critical molecules whose dysfunction can have detrimental impacts on the network’s function. More advanced applications of molecular fault diagnosis engineering in target discovery and drug development are discussed in [3] and [4].

In this study, the basic molecular fault diagnosis approach introduced in [2] is expanded in a number of ways. First, different levels for fault probability are introduced for each molecule, which are real numbers between 0 and 1. This allows the network vulnerabilities to be parameterized using a parameter that changes in a continuous way between 0 and 1. Then a method for computing the vulnerabilities of molecules based on the continuous fault probabilities is developed. Moreover, the impact of different combinations of input activities on the activities of the output molecules and also the levels of molecular vulnerabilities are examined. Since Abdi et al. [2] assumed that only one molecule can be faulty at a given time, in this study we expand this approach to scenarios where two molecules are simultaneously faulty. We compute the vulnerability level for each pair of molecules, to understand how simultaneous faulty states of two molecules can contribute to the malfunction of the network. Another assumption considered by Abdi et al. [2] was the binary activity model for molecules, i.e., a molecule could be either active or inactive. This modeling approach has been used over years, to characterize different types of networks; including signaling networks (see, for example, the review articles [5]–[8]). Here we extend the fault diagnosis technique by considering three activity levels, i.e., active, partially active, and inactive states, and then compute molecular vulnerability levels for the ternary case. This allows to evaluate the effect of having more than two activity states on the computed vulnerabilities.

## Methods

**Figure 1:**
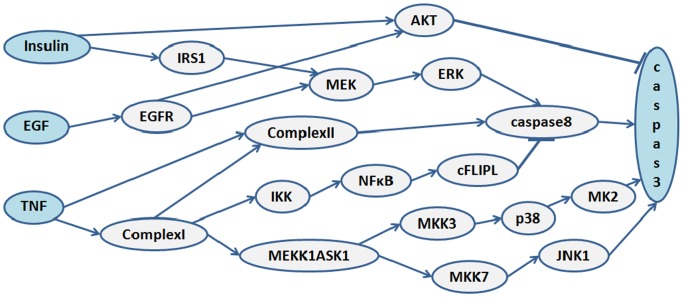
The caspase3 network. The three input molecules are insulin, EGF and TNF, which regulate the output molecule, caspase3, via some intermediate molecules.



Caspase3 is a well characterized molecule that is critically involved in cell death and cell survival. Several upstream signaling pathways, typically originated from the ligands EGF, epidermal growth factor, insulin and TNF (tumor necrosis factor), merge to regulate the activity of caspase3 (Figure 1). This network is selected for this study because it has been extensively characterized and experimentally verified by several independent groups of scientists, and the experimental data of the activity levels of the molecules measure under different conditions are available for the comparisons with the developed methods in this study [9]. There are seventeen intermediate molecules between the inputs and the output. To analyze this network, we define the transition probability matrix, which basically characterizes the network input-output functional relations. According to Figure 1 and the input-output relationships in Table 1 of [2], constructed using the experimental findings of [9], the network transition probability matrix **M** can be written as:

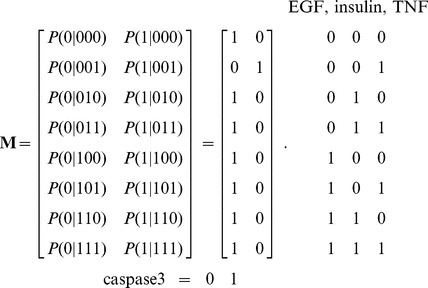
(1)Each element in the above matrix is a conditional transition probability of the form 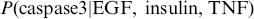. For any given set of 0/1 values for the inputs shown in (1), the matrix specifies the probability of the output to be 0 or 1. For example, according to the input-output relationships in Table 1 of [2], when EGF and insulin are active and TNF is inactive, capsase3 becomes inactive, i.e., 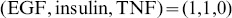 results in 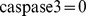. This implies that 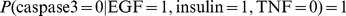 and 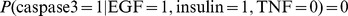. Note that the network matrix **M** in (1) agrees with the experimental findings of [9]. Equations for the activity of each molecule in terms of its input signals are provided in Table S1.

When a molecule becomes faulty, due to mutations or some other abnormalities, its activity level does not change in the network, irrespective of its inputs signals [2]. Note that a dysfunctional molecule is considered to be inactive, 0, and its state remains unchanged, no matter what the states of its regulatory inputs are. Since decreased expression or activity of molecules occur in many human diseases, in this paper we focus on the 0 status, reflecting the hypoactivity of a molecule. However, increased activity or abundance of some signaling molecules are reported in several diseased conditions as well. In such a scenario, the same methods developed here can be repeated considering the 1 status, reflecting hyperactivity of a molecule. A faulty molecule in the network changes the network matrix **M**. Assume the probability of a molecule 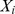 in the network to be faulty is *p*, i.e., 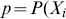 is faulty). The caspase3 network matrix **M** can be constructed using the equations in Table S1, by calculating the conditional probabilities specified in (1). Depending on the faulty molecule, the network matrix can take different forms. For the caspase3 network we have observed that there are four different network matrices: when AKT is faulty, **M** is given in (2); for a faulty EGFR, **M** is provided in (3); a faulty MEKK1ASK1 results in the **M** presented in (4); and when other molecules are faulty, **M** takes the form given in (5). Note that when AKT is faulty, the faulty network matrix in (2) is very different from the normal matrix in (1). For EGFR and MEKK1ASK1, the faulty network matrix differs from the normal matrix only in one row. The network matrix does not change, when others molecules are faulty. In what follows, we compute the vulnerability level of each molecule in the network using the faulty network matrices given in (2–5).
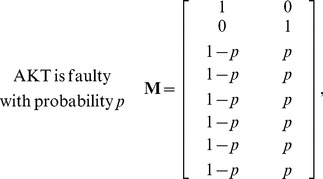
(2)




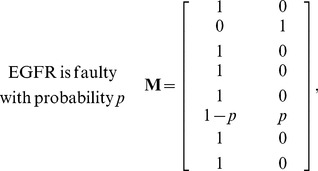
(3)




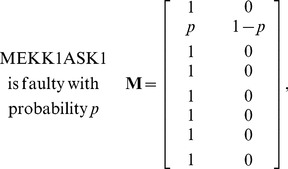
(4)




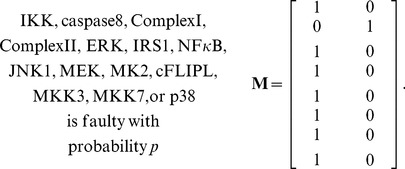
(5)


### Vulnerabilities of molecules in the caspase network

By definition, vulnerability level of a molecule in a network is the probability that the network fails (does not provide the expected output), when that molecule is dysfunctional [2]. The vulnerability of a network to the dysfunction of each individual molecule can be computed as follows, using the total probability theorem [10]:
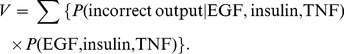
(6)


For eight equi-probable patterns of 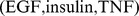, the vulnerability formula in (6) can be written as:
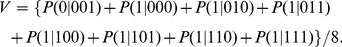
(7)


The above conditional probabilities 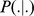 are elements of the faulty network matrices 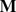 in (2–5). By substituting the elements of the matrices of (2–5) into (7), the following equations can be obtained for the vulnerability of each molecule in terms of its fault probability *p*:
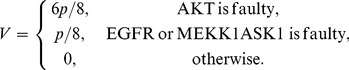
(8)


**Figure 2:**
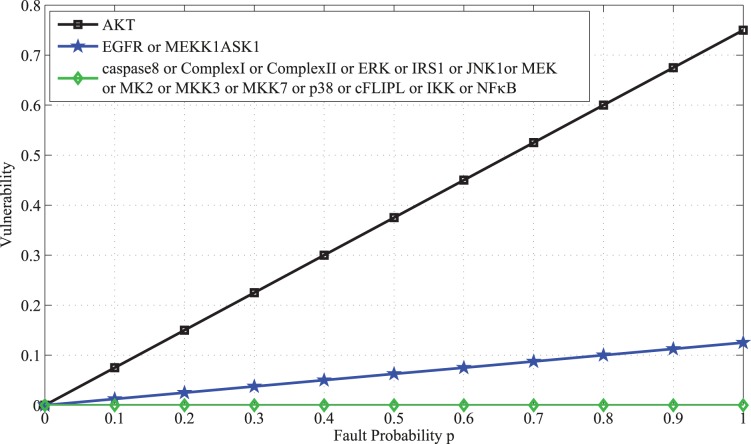
Vulnerability versus the fault probability *p* of each molcule in the caspase3 network. Vulnerability, which is the probability of the network function failure, shows a non-decreasing trend as the fault probability of a molcule increases. Vulnerability is the highest when AKT is faulty (black graph). When EGFR or MEKK1ASK1 is faulty, vulnerability is the same (blue graph), but less than AKT’s vulnerability. Vulnerability is zero (green graph), when each of the rest of the molecules is faulty.

The above results are plotted in Figure 2. Based on the above results, one can categorize the molecules in the network into three groups: a highly vulnerable molecule (AKT), molecules with low vulnerabilities (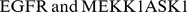) and molecules with zero vulnerabilities (the rest of the molecules). This is further discussed in the Results and Discussion sections.

### Impact of input activities on the output activity

#### Output activity with no faulty molecule in the network

Each input activity represents the probability of a ligand binding to its receptor on the cell membrane. Beginning from the case where all the molecules are functioning normally, we study the impact of input activities on the output activity. Let the parameters 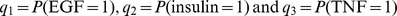 be the input activities. Based on the experimentally-verified caspase network input-output relations provided in Table 1 of [2] we have:
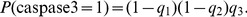
(9)


**Figure 3:**
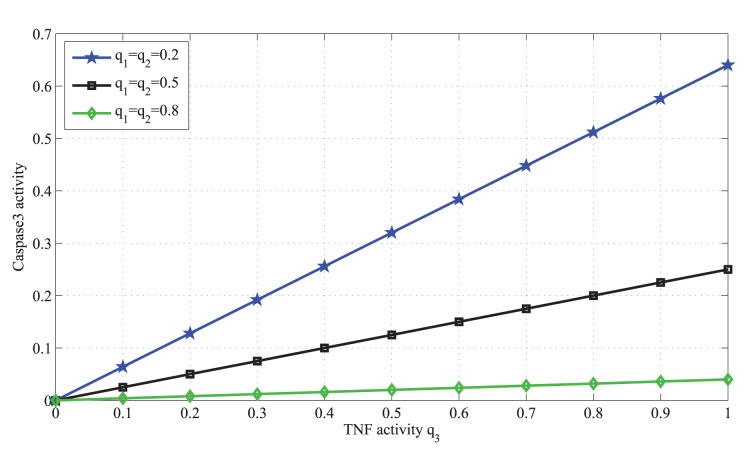
Caspase3 activity in terms of the TNF activity 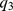. In this figure the activity of TNF changes from 0 to 1, whereas the activities of EGF and insulin are the same, 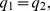 both fixed at 0.2 (blue graph), 0.5 (black graph), and 0.8 (green graph). Overall, caspase3 activity increases with TNF activity. However, its activity decreases as EGF and insulin become more active.

This equation is plotted in Figure 3 and explained in Results.

#### Output activity with one faulty molecule in the network

When there is one faulty molecule in the network, the caspase3 activity can be calculated using the total probability theorem:
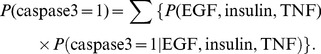
(10)


The term 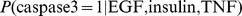 can be obtained from the second column of the network matrices in (2–5).

To see how the activity of a faulty molecule may affect the output activity, when an input activity changes, consider the example in which 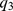, the activity of the input TNF changes from 0 to 100%, whereas similarly to [2], activities of the other two inputs, EGF and insulin, are fixed at 50%, i.e., 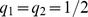. Using (10), the output activity can be written in terms of 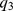:
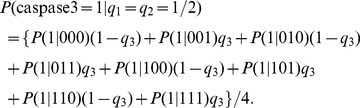
(11)


As discussed previously, the parameters 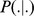 in the above formula depend on the faulty molecule we are studying. As some examples, here we examine the output activity when the faulty molecule has high vulnerability (AKT), low vulnerability (MEKK1ASK1) or zero vulnerability (IKK).

*a) The faulty molecule is highly vulnerable:* When AKT is faulty with probability *p*, by replacing the parameters 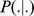 in (11) with the second column of the network matrix in (2), the output activity can be written as:
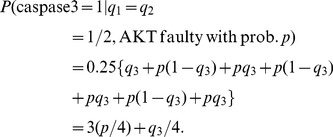
(12)


*b) The faulty molecule has low or zero vulnerability:* When MEKK1ASK1 or IKK is faulty with probability *p*, by replacing the parameters 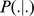 in (11) with the second columns of the network matrices in (4) or (5), respectively, the output activity can be written as follows:
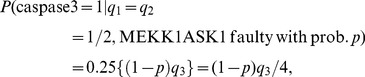
(13)




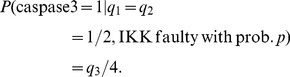
(14)


**Figure 4:**
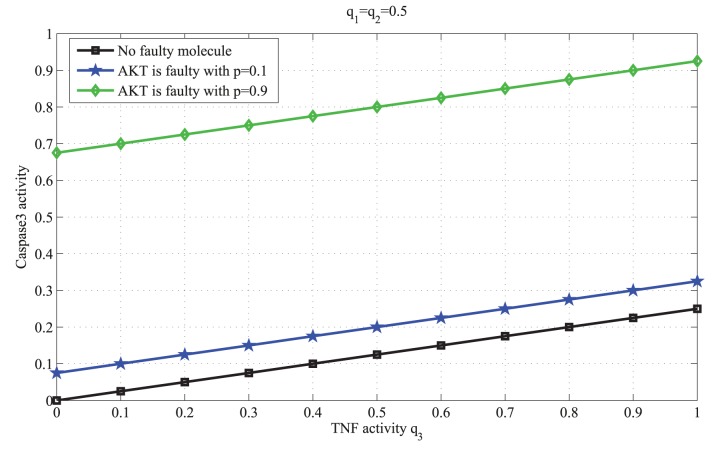
Caspase3 activity in terms of the TNF activity 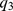, when AKT is faulty. In this figure the activity of TNF changes from 0 to 1, whereas the activities of EGF and insulin are the same, 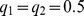. As a baseline, the black graph shows the output activity when there is no faulty molecule. When AKT’s fault probability is small, 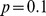, the output activity slightly increases (blue graph). However, when AKT’s fault probability is large, 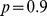, the output activity increases significantly (green graph).

**Figure 5:**
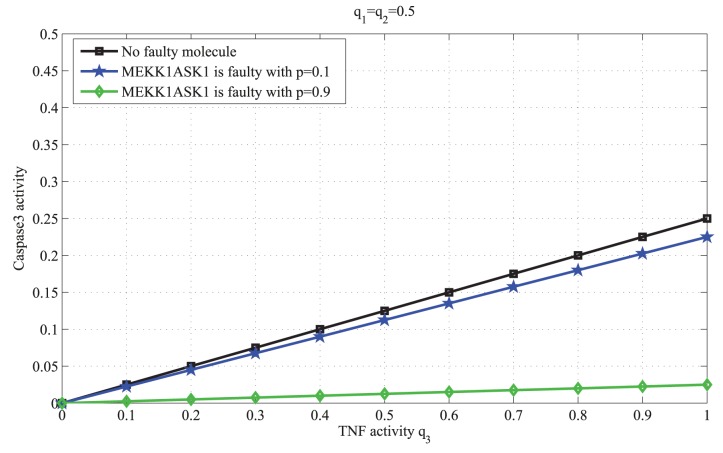
Caspase3 activity in terms of the TNF activity 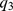, when MEKK1ASK1 is faulty. In this figure the activity of TNF changes from 0 to 1, whereas the activities of EGF and insulin are the same, 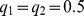. As a baseline, the black graph shows the output activity when there is no faulty molecule. When MEKK1ASK1’s fault probability is small, 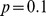, the output activity slightly decreases (blue graph). However, when MEKK1ASK1’s fault probability is large, 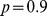, the output activity decreases significantly (green graph).

**Figure 6:**
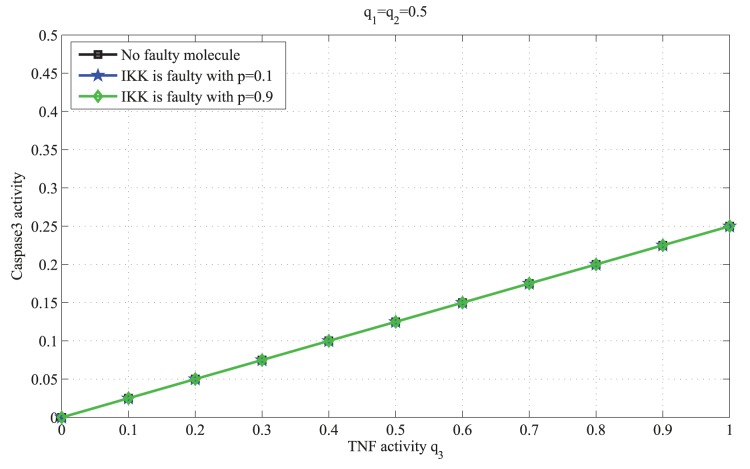
Caspase3 activity in terms of the TNF activity 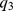, when IKK is faulty. In this figure the activity of TNF changes from 0 to 1, whereas the activities of EGF and insulin are the same, 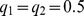. We observe that the output activity does not change, whether IKK is faulty or not. This is because IKK’s vulnerability is zero (as shown in Methods).

Equations (12–14) are graphed in Figure 4, Figure 5 and Figure 6, respectively, and their biological implications are discussed in the Results section.

### Vulnerabilities of pairs of faulty molecules

To compute network vulnerabilities, we originally assumed a single faulty molecule is present in the network at a given time. Now we study the case where two molecules become simultaneously faulty. Let 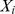 and 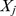 represent two simultaneously faulty molecules, such that they are both stuck at 0 (sa0). Therefore we have 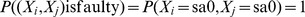. Biologically, this means that two molecules remain inactive, irrespective of their input signals. By calculating the conditional probabilities specified in (1) for all the double faults in the network, network matrices for all the double faults are constructed and listed in Equations (S1) - (S8). By substituting the elements of these matrices into (7), network vulnerabilities for all possible double faulty molecules are computed and listed in Table S2, for equi-probable inputs. Sorted joint vulnerabilities, from the highest to lowest values, are given in Table S3. A summary of Table S2 is provided in Table S4, which includes the average of all vulnerabilities associated with each molecule, when it is jointly faulty with other molecules. Note that the diagonal elements of Table S2 are single fault vulnerabilities, which are obtained by substituting 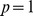 in (8).

### Ternary fault diagnosis

In our previously published papers, we considered two fundamental activity states of molecules, i.e., active or inactive states. Now we propose a ternary activity model, where a molecule could be active, partially active or inactive, represented numerically by 1, 1/2 and 0, respectively. This modeling scenario is biologically relevant because in several disease conditions the activity or the protein levels of molecules are partially affected [7]. For example, phosphorylation of AKT at Thr-308 is required for its activity, whereas the second phosphorylation at Ser-473 can make the molecule more active [11]. In what follows, we develop a ternary molecular fault diagnosis method.

#### The developed ternary fault diagnosis method

The first step is to write the input-output relationships for each molecule in the network in a ternary format. This is because there are different levels of activity in the network, i.e., inactive, partially active and active states for each molecule, represented numerically by 0, 1/2 and 1, respectively. Using ternary logic [12], an equation for the activity of each molecule in terms of its input signals is derived (Table S5). Using these input-output relations, the network matrix M for the ternary model is computed and presented in Equation (S9).

Now we introduce a faulty network ternary model for the caspase3 network, to analyze the impact of dysfunctional molecules. Suppose the probability of a molecule 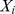 in the network to be faulty is *p*, i.e., 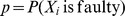. When a molecule is faulty, its activity state does not change in response to its regulators, and gets stuck at 0. This causes the network matrix in (S9) to change. The faulty network matrix can be constructed by calculating the conditional probabilities specified in (S9). Depending on the faulty molecule, the network matrix in the ternary case can take six different forms, as listed in (S10–S15). In what follows, we compute the vulnerability level of each molecule in the network using the faulty network matrices given in (S10–S15).

For twenty seven equi-probable ternary patterns of (EGF, insulin, TNF), the vulnerability formula in (6) can be written as:
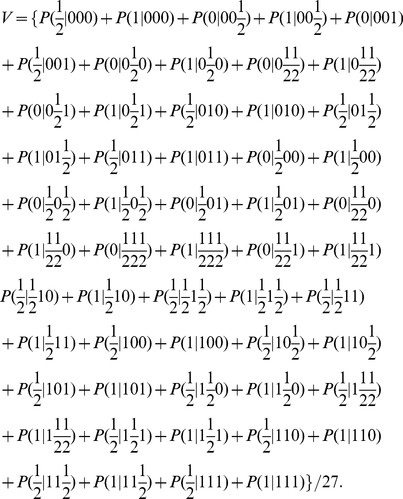
(15)


The above conditional probabilities are elements of the faulty network matrices 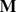 in (S10–S15). By substituting those elements into (15), the following equations are obtained for the vulnerability of each molecule in terms of its fault probability *p*:
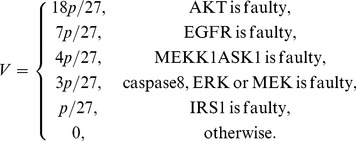
(16)


**Figure 7:**
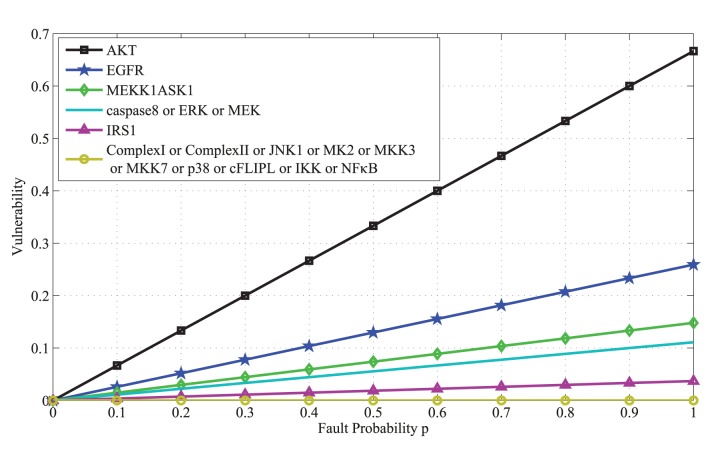
Vulnerability versus the fault probability *p* in the caspase3 network, copmuted using a ternary activity model. Upon considering three levels of activity for each molecule, active, partially active, and inactive, vulnerability of each molecule is graphed in terms of *p*. We observe that in the caspase3 network molecules are categorized into six groups, according to their vulnerability levels. Vulnerability is still the highest when AKT is faulty (black graph). EGFR vulnerability (blue) is higher than MEKK1ASK1 vulnerability (green). Vulnerabilities of the rest of the molecules are all below 0.1.

The above results are plotted in Figure 7 and discussed in Results.

## Results

In Figure 2 the vulnerability levels of molecules in the caspase3 network (Figure 1) are plotted in terms of the fault probability *p*, using Equation (8). The figure reveals different fault behaviors in the network, depending on the faulty molecule. According to the figure, AKT has an important role in the function of the network. When AKT is faulty, the network vulnerability rapidly increases with *p*. On the other hand, the vulnerability is low and also less sensitive to the changes in *p* for some molecules such as EGFR and MEKK1ASK1. This means that their role is less critical to the network function. For the rest of the molecules, the vulnerability is zero. This finding is biologically relevant and in agreement with the fact that molecular networks typically have high redundancy and the function of signaling molecules are often compensable through different mechanisms [2] [13].

### Input-output activity relashionships in the presence or absence of a faulty molecule

First we consider the case where there is no faulty molecule in the network. Based on Equation (9), the graphs in Figure 3 show how the capase3 activity changes with the input activities. When activities of EGF and insulin are fixed at a certain level, increasing the activity of TNF makes caspase3 more active. However, for a fixed TNF activity, increasing the activity of EGF and insulin reduces the activity of caspase3. These results are consistent with the experimental data [9], i.e., activation of caspase3 by TNF and the subsequent cell death are inversely correlated with the activity of EGF and insulin.

To study possible impacts of faulty molecules on the output molecule, we consider molecules with different vulnerability levels. According to Equation (12), when AKT, a highly vulnerable molecule, is faulty, we observe that the output activity can significantly change (Figure 4), depending on the fault probability of AKT. This is biologically relevant, since the activity of AKT has a positive correlation with caspase3 activity [11]. On the other hand, based on Equation (13), when a molecule with low vulnerability such as MEKK1ASK1 is faulty, it makes only some small changes to the output (Figure 5). When a molecule such as IKK whose vulnerability is zero becomes faulty, Equation (14), no change in the output activity is observed (Figure 6). This is because when IKK is faulty, there are other molecules and pathways in the network that allow the output to be properly regulated by the input signals. In other words, the activity of IKK is readily compensable in this network, whereas the activity of AKT is required for propagation of input signals, to correctly regulate the output activity.

### The effect of input activities on vulnerabilities

**Figure 8:**
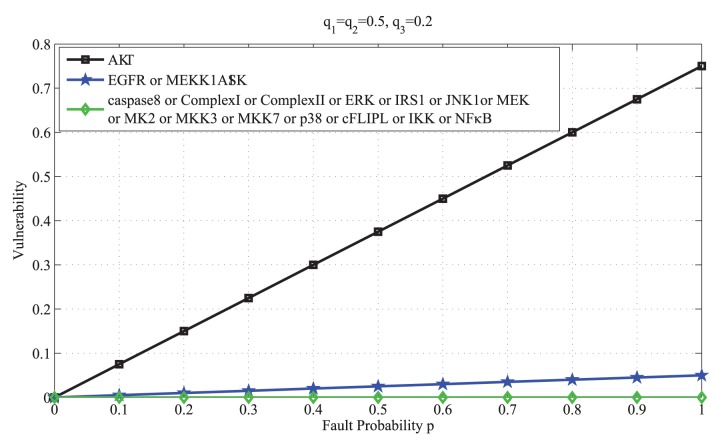
Vulnerability versus the fault probability *p* for all the molecules in the caspase3 network, while TNF activity is low. Here TNF activity is 0.2, whereas EGF and insulin activities are fixed at 0.5. We observe that the vulnerability of AKT rapidly increases with *p*, whereas the vulnerability of other molecules are almost zero. This indicates the critical role of AKT in the network.

**Figure 9:**
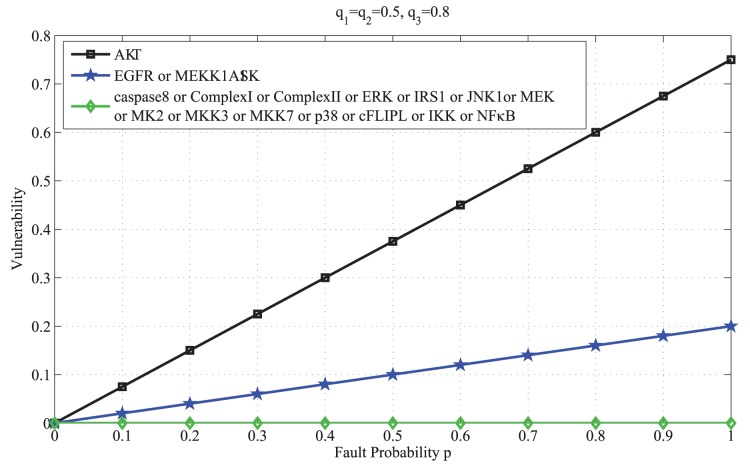
Vulnerability versus the fault probability *p* for all the molecules in the caspase3 network, while TNF activity is high. Here TNF activity is 0.8, whereas EGF and insulin activities are fixed at 0.5. We observe that the vulnerability of AKT rapidly increases with *p*, whereas the vulnerabilities of other molecules are zero, except for EGFR and MEKK1ASK1. Due to the increased activity of TNF, these two molecules show some level of vulnerability, which was not present when TNF activity was low.

In Supporting Information, Equation (S17) that relates vulnerabilities to the input activities is derived. The results are graphed in Figure 8 and Figure 9, to show how the vulnerability of a molecule may depend on the inputs’ activity levels. When TNF activity is low, vulnerability of AKT rapidly increases with the fault probability *p*, whereas the vulnerability of other molecules is almost zero (Figure 8). This indicates the critical role of AKT in the network. When the activity of TNF is increased, we observe that the vulnerability of AKT still rapidly increases with *p*, whereas the vulnerabilities of other molecules are zero, except for EGFR and MEKK1ASK1 (Figure 9). Due to the increased activity of TNF, these two molecules now show some level of vulnerability, which was not present when TNF activity was low.

### Vulnerability levels of pairs of faulty molecules

So far we have discussed the case where there is only one faulty molecule in the network, to understand the role of each individual molecule in the failure of network. Now we discuss the results we have obtained for a more complex scenario, where two molecules in the network become simultaneously dysfunctional. Vulnerability levels for all pairs of molecules are provided in Table S2. To better comprehend the results, all pairs are sorted in Table S3 in a descending order, according to their vulnerability levels. Analysis of double faults shows that a pair with high vulnerability typically includes one molecule that has high vulnerability, individually. In the caspase3 network, we notice that one of the molecules of the pairs with high double fault vulnerability level is AKT. As shown above, in this network AKT exhibits the highest vulnerability level in the single fault analysis scenario (see Equation (8) and Figure 2). Additionally, we observe that if a molecule has a low vulnerability level in the single fault model, it typically exhibits low vulnerability levels when paired with other low vulnerable molecules.

To further investigate double fault vulnerabilities, we consider some examples. In Table S6 we have listed the network output, for all different input combinations, when faulty molecules are AKT or p38 or MEKK1ASK1, as well as the pairs of (AKT, p38) or (AKT, MEKK1ASK1). Note that faulty (incorrect) outputs are marked in italic bold. When AKT is faulty (stuck at 0 or sa0), the number of incorrect outputs is 6, which results in the vulnerability of 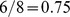 for AKT. Also when AKT is jointly faulty with p38, the number of incorrect outputs is 6, which gives the vulnerability of 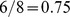 for the pair of (AKT, p38). On the other hand, when AKT and MEKK1ASK1 are jointly faulty, the number of incorrect outputs in Table S6 becomes 4, which indicates the vulnerability of 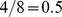. So, depending on what pairs are dysfunctional, the vulnerability level of each pair could be different.

Another way of analyzing double faults is to look at the average of all the vulnerabilities associated with a molecule, when it is jointly faulty with other molecules (Table S4). Vulnerabilities for single faulty molecules are also provided in Table S4 for comparison. We notice that if a molecule has a low single fault vulnerability, on average it exhibits small double fault vulnerabilities.

### Ternary fault diagnosis and comparison with binary fault diagnosis

**Figure 10:**
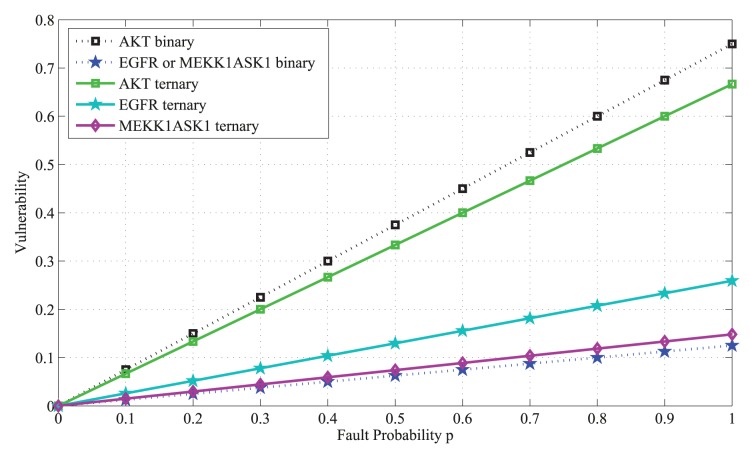
Comparing vulnerabilities in the caspase3 network obtained via binary and ternary network models. In this figure dashed and solid graphs represent vulnerabilities for binary and ternary activity models, respectively. In both models AKT shows high vulnerability. While in the binary model EGFR and MEKK1ASK1 exhibit the same vulnerablity, the ternary model shows somewhat different yet still low vulnerability for EGFR. Since vulnerablities for the rest of the molcules are very low in both models, they are not shown, to keep the figure easy to read.

Here we study the network fault behavior when each molecule has three levels of activity. Using Equation (16), vulnerabilities of different molecules are graphed in Figure 7. This study helps in understanding how the prediction power changes, when the more complex ternary model and equations are used for fault diagnosis. Similarly to the binary activity case, vulnerabilities increase with the fault probability *p*. Additionally, AKT still persistently shows the highest vulnerability in the network. Compared to the binary case (Equation (8) and Figure 2), in ternary model we observe more resolution in the vulnerability values of molecules with lower vulnerabilities, which is reflected by the higher number of molecules with separate graphs in Figure 7. To investigate this matter, in Figure 10 ternary and binary vulnerabilities are graphed together, using Equations (8) and (16). For a highly important molecule such as AKT, transition from binary to the more complex ternary model results in a slight change in its vulnerability. For some molecules such as EGFR and MEKK1ASK1, we observe slightly higher yet still small vulnerability levels in the ternary model, compared to the binary model (Figure 10). Vulnerabilities of the rest of the molecules have either changed slightly in the ternary model or remained to be zero, compared to the binary model. Their vulnerability values all fall below 0.1 and are not graphed in Figure 10, to keep the figure easy to read.

### Analysis of SHP2 signaling network

**Figure 11:**
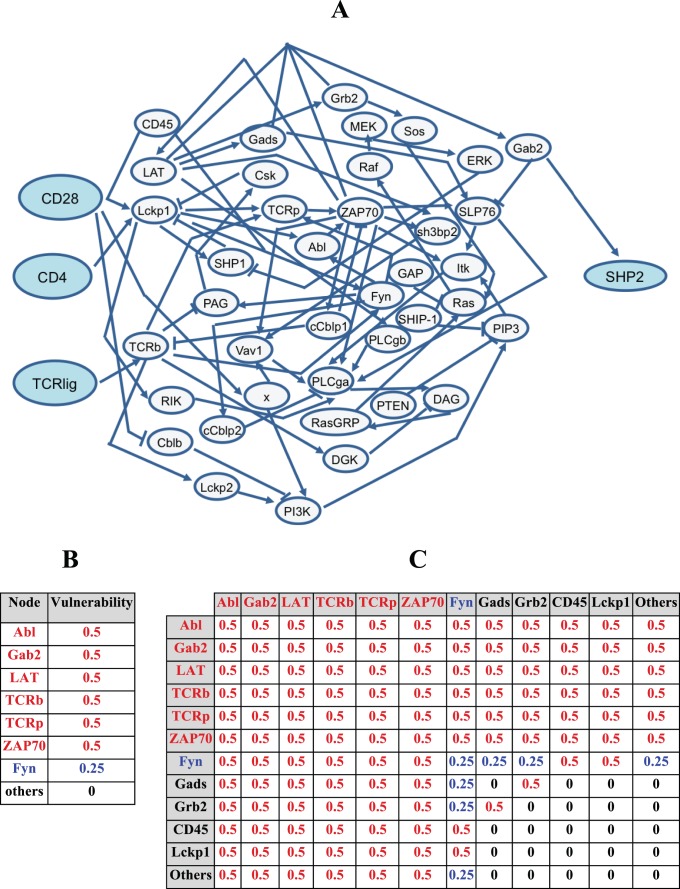
The SHP2 network. (A) The three input molecules are TCRlig, CD4 and CD28, which regulate the output molecule SHP2. (B) Network vulnerabilities for single faults in the SHP2 network. Highly vulnerable molecules are marked in red, blue is used to identify molecules with low vulnerability and molcules with zero vulnerability are shown in black. (C) Network vulnerabilities for all pairs of faulty molecules (the diagonal elements of the table are single fault vulnerabilities). The color code is the same as panel B.

We have also analyzed the experimentally-verified model of SHP2 network [14] using the methodology that is proposed and developed in this study. This large network is composed of three input molecules and many intermediate molecules that regulate the output molecule SHP2 (Figure 11A). It has about seventy interactions and multiple feedback loops [14]. Equations for the activity of each molecule in terms of its input signals are provided in Table S7. SHP2 is a nonreceptor phosphatase that is expressed in every tissue. SHP2-mediated Ras-ERK1/2-MAP kinase pathway is involved in regulation of cell survival, proliferation, differentiation, adhesion and migration, depending on cell contexts. Targeting the activity of SHP2 and other tyrosine phosphatases is a novel strategy used in anticancer drug discovery [15].

Analysis of single faults in the SHP2 network (Figure 11B) shows that Abl, Gab2, LAT, TCRb, TCRp, and ZAP70 are highly vulnerable molecules, Fyn has low vulnerability, and the rest of the molecules have zero vulnerability. The critical roles of some of these highly vulnerable molecules in cancer are well documented. Abl is a protein tyrosine kinase that is not only critically involved in the progression of many types of malignancies, but also its inhibitor Gleevec, also known as Imatinib, is considered as a breakthrough in targeted therapy of cancer [16]. Gab2 is a scaffolding protein that contains various structural domains and docking sites that serve as a platform for the assembly of signaling systems. Gab2 contributes to an invasive and metastatic phenotype in breast carcinogenesis and is known as a potential therapeutic target in cancer therapy [17].

Double fault analysis of the SHP2 network (Figure 11C) demonstrates that a pair with zero vulnerability typically includes two molecules that have zero vulnerabilities, individually. However, a pair with at least one highly vulnerable molecule exhibits high vulnerability (Figure 11C). These results agree with the double fault analysis of the caspase network.

There are some cases that deserve further discussion. Grb2 and Gads both have low vulnerabilities, individually. However, when they are jointly faulty, the pair shows high vulnerability. This can be explained as follows: According to Table S7, Gab2(t+1) = LAT(t) & ZAP70(t) & (Gads(t) * Grb2(t)), where & and * stand for AND and OR, respectively. The equation means that for Gab2 to be active at the time instant t+1, LAT and ZAP70 and either Gads or Grb2 have to be active at the previous time instant t. When Grb2 and Gads are jointly faulty, stuck at 0, Gab2 remains inactive regardless of the states of LAT and ZAP. According to the equation we have Gab2(t+1) = LAT(t) & ZAP70(t) & (0*0) = LAT(t) & ZAP70(t) & 0 = 0. Since Gab2 is the sole activator of SHP2, SHP2 remains inactive when both Grb2 and Gads are stuck at 0. Under these circumstances and according to our calculations, for four out of eight different input combinations, the activity of SHP2 is different from its normal states, which results is the vulnerability of 0.5 for the faulty pair of Grb2 and Gads. Another similar case is the highly vulnerable pair of Fyn and CD45, where none of them are highly vulnerable, individually. Similarly to the previous case, this can be explained using the equations in Table S7. In biological terms, many complex trait disorders are reported to be associated with the simultaneous dysfunction of more than one gene. In the Discussion Section below, we provide the example of schizophrenia, where simultaneous dysfunction of a few signaling molecules are reported to be associated with the development of pathology [18], [19].

## Discussion

In this study, more advanced fault analysis methods are developed and applied to caspase and SHP2 networks. We have analyzed the networks under different assumptions and conditions. In the first fault analysis paper [2], we considered the case where there is only one single faulty molecule in the network at a given time. Here we have extended the work by considering pairs of simultaneously faulty molecules, and have developed a method for calculating network vulnerabilities to the dysfunction of pairs of molecules. The results indicate that high double fault vulnerabilities in the casepase network are for cases where AKT is one of the faulty molecules. To further analyze double faults, we have computed the average of all the vulnerabilities associated with a molecule, when it is jointly faulty with other molecules in the network. It is observed that if a molecule has a low single fault vulnerability, on average it exhibits small double fault vulnerabilities.

Analysis of double faults in a more complex network such as the SHP2 network provides further interesting results. In the SHP2 network, Grb2 and Gads both have low vulnerabilities, individually. However, when they are jointly faulty, the pair shows high vulnerability. Although certain experiments are necessary to confirm this specific finding empirically, this particular example shows that changes in the activity of a single molecule may easily be compensated and tolerated by the network. However, when it is accompanied by changes in the activity of another molecule involved in the regulation of the same output, then the compensatory mechanisms may not be sufficient to overcome the failure of the network. Analysis of double and multiple faults in molecular networks is particularly important because they can be used to model complex trait disorders. Complex trait disorders are resulted from the dysfunction of multiple genes. The most common human disorders are in fact complex trait disorders. In a wide variety of complex human disorders, including cancer, metabolic disorders such as diabetes, neurological disorders such as Alzheimer’s disease, and psychiatric disorders such as schizophrenia, depression and addiction, the critical roles of several signaling molecules are consistently reported by multiple groups of independent scientists. For example, in a complex disorder such as schizophrenia, it has been consistently reported that several specific signaling molecules, including AKT, DISC1, NRG1 and calcineurin, are associated with the disease [18] [19] [20]. Moreover, a variety of neurotransmitter systems that are heavily regulated through intracellular signaling networks, including glutamate, dopamine, serotonin and GABA neurotransmitter systems, have been reported for many decades to play a critical role in the pathogenesis of schizophrenia.

Review of the literature published during the past few years provides strong support for the involvement of AKT/GSK3 signaling pathway in the development of schizophrenia. A number of studies strongly suggest that targeting this pathway is a promising approach for development of novel psychotropic drugs for treatment of schizophrenia and mood disorders. At the same time, there are several other signaling molecules and pathways that show conclusive evidence for being involved in the pathogenesis of schizophrenia. Despite many differences in the approach of scientists working on schizophrenia, they have come to a general consensus that schizophrenia is most likely caused by the altered function or expression of many genes. Such genes may individually contribute only to a small risk, but their cumulative effects cause the dysfunction of brain, which manifests itself by the clinical picture we call schizophrenia [20]. Although there has been significant progress in identification of the role of several signaling molecules in schizophrenia, we still do not know how much each gene contributes to the development of pathology. Therefore, we need new systems biology tools that can quantify the role of individual or multiple genes in disease development. The recently developed fault diagnosis engineering technology for molecular networks is a promising tool that has such capabilities and can model complex trait disorders such as schizophrenia [18]. The double fault model presented in this study can be extended to a multi-fault model, in which simultaneous dysfunction of several genes involved in schizophrenia could be studied. The presented fault diagnosis approach can model a complex trait disorder such as schizophrenia because it can quantify the role of each individual gene, pairs of genes, as well as the combination of multiple genes known to be involved in the pathogenesis of this complex trait disorder [19].

In the previous fault analysis paper [2] we studied the case where each molecule had an active or inactive state. Here we have expanded the approach by considering three levels of activity for each molecule, and have developed a method for calculating network vulnerabilities for the ternary model. Our results for the caspase network show that predictions obtained using the more complex ternary model are about the same as the predictions of the simpler binary approach. Our results suggest that for the purpose of fault diagnosis it is more practical to start with the less complex active/inactive fault diagnosis approach, to analyze the malfunction of signaling networks. This assists in identifying many molecules whose dysfunction do not contribute to the network failure (molecules with low vulnerability) [2]. Afterwards, if one may want to further study the role of molecules with medium or high vulnerabilities, he can focus on building a less complex model where only the small set of such molecules have three activity levels. Overall, the important conclusion is that by increasing the number of activity levels for each molecule, the complexity of the model and its fault analysis significantly increases. However, the predictive power of the model does not necessarily appear to increase proportionally.

There have been some recent studies on tristability in genetic networks [21] [22]: It is shown that the microRNA-transcription factor self-activating chimera toggle switches can exhibit three metastable states [21], whereas the microRNA/ZEB ternary switch is shown to result in three phenotypes [22]. Our ternary network analysis, however, is different from these studies. We have focused on signaling networks with ligands as inputs and some molecules as outputs, and have considered three activity levels for each molecule. Our goal is to determine the vulnerability of the network to the possible dysfunction of its molecular components. This research goal is different from those considered in [21] and [22], and the methodology developed here addresses a different problem.

## Supporting Information

Table S1 Binary Equations for the Caspase3 Network.
(DOCX)

Table S2 Network Vulnerabilities for All Pairs of Faulty Molecules in the Caspase3 Network.
(DOCX)

Table S3 Sorted Network Vulnerabilities for All Pairs of Faulty Molecules in the Caspase3 Network.
(DOCX)

Table S4 Average of All Double Fault Vulnerabilities Associated with each Molecule, when Simultaneously Faulty with Other Molecules in the Caspase3 Network.
(DOCX)

Table S5 Ternary Equations for the Caspase3 Network.
(DOCX)

Table S6 Network Output for Some Single and Double Faulty Molecules in the Caspase3 Network (Incorrect Outputs Are Italic and Bold).
(DOCX)

Table S7 Binary Equations for the SHP2 Network.
(DOCX)

Methods S1 (DOCX)
