# Supplementary material for: Advanced Fault Diagnosis Methods in Molecular Networks
Source: PLoS One. 2014 Oct 7;9(10):e108830. doi: 10.1371/journal.pone.0108830 (PMC4188586; doi:10.1371/journal.pone.0108830)
Supplement: Table S1 — Binary Equations for the Caspase3 Network. (DOCX) [file pone.0108830.s001.docx]

**Table S****1:**  Binary Equations for the Caspase3 Network in Figure 1

|  | Molecules | Binary equations |
| --- | --- | --- |
| Molecules in the network (listed  alphabetically) | AKT | AKT = EGFR \| insulin |
|  | caspase8 | caspase8 = (~cFLIP_L_) & (ComplexII \| ERK) |
|  | cFLIPL | cFLIPL = NFκB |
|  | ComplexI | ComplexI = TNF |
|  | ComplexII | ComplexII = TNF \| ComplexI |
|  | EGFR | EGFR = EGF |
|  | ERK | ERK = MEK |
|  | IKK | IKK = ComplexI |
|  | IRS1 | IRS1 = Insulin |
|  | JNK1 | JNK1 = MKK7 |
|  | MEK | MEK = EGFR \| IRS1 |
|  | MEKK1ASK1 | MEKK1ASK1 = ComplexI |
|  | MK2 | MK2 = p38 |
|  | MKK3 | MKK3 = MEKK1ASK1 |
|  | MKK7 | MKK7 = MEKK1ASK1 |
|  | NFκB | NFκB = IKK |
|  | p38 | p38 = MKK3 |
| Network output molecule $\to$ | caspase3 | caspase3 = (~AKT) & (caspase8 \| JNK1 \| MK2) |

Each equation specifies the input signal(s) of a molecule in the network. The binary operations ~, | and & represent NOT, OR and AND, respectively [2].
