# Supplementary material for: Advanced Fault Diagnosis Methods in Molecular Networks
Source: PLoS One. 2014 Oct 7;9(10):e108830. doi: 10.1371/journal.pone.0108830 (PMC4188586; doi:10.1371/journal.pone.0108830)
Supplement: Table S2 — Network Vulnerabilities for All Pairs of Faulty Molecules in the Caspase3 Network. (DOCX) [file pone.0108830.s002.docx]

**Table S2:** Network Vulnerabilities for All Pairs of Faulty Molecules

|  | AKT | caspase8 | cFLIPL | ComplexI | ComplexII | EGFR | ERK | IKK | IRS1 | JNK1 | MEK | MEKK1ASK1 | MK2 | MKK3 | MKK7 | NFκB | p38 |
| --- | --- | --- | --- | --- | --- | --- | --- | --- | --- | --- | --- | --- | --- | --- | --- | --- | --- |
| AKT | 0.75 | 0.375 | 0.75 | 0.75 | 0.75 | 0.625 | 0.375 | 0.75 | 0.625 | 0.75 | 0.375 | 0.5 | 0.75 | 0.75 | 0.75 | 0.75 | 0.75 |
| caspase8 | 0.375 | 0 | 0 | 0.125 | 0 | 0.125 | 0 | 0 | 0 | 0 | 0 | 0.125 | 0 | 0 | 0 | 0 | 0 |
| cFLIPL | 0.75 | 0 | 0 | 0 | 0 | 0.125 | 0 | 0 | 0 | 0 | 0 | 0 | 0 | 0 | 0 | 0 | 0 |
| ComplexI | 0.75 | 0.125 | 0 | 0 | 0.125 | 0.125 | 0 | 0 | 0 | 0 | 0 | 0 | 0 | 0 | 0 | 0 | 0 |
| ComplexII | 0.75 | 0 | 0 | 0.125 | 0 | 0.125 | 0 | 0 | 0 | 0 | 0 | 0.125 | 0 | 0 | 0 | 0 | 0 |
| EGFR | 0.625 | 0.125 | 0.125 | 0.125 | 0.125 | 0.125 | 0.125 | 0.125 | 0.125 | 0.125 | 0.125 | 0.125 | 0.125 | 0.125 | 0.125 | 0.125 | 0.125 |
| ERK | 0.375 | 0 | 0 | 0 | 0 | 0.125 | 0 | 0 | 0 | 0 | 0 | 0.125 | 0 | 0 | 0 | 0 | 0 |
| IKK | 0.75 | 0 | 0 | 0 | 0 | 0.125 | 0 | 0 | 0 | 0 | 0 | 0 | 0 | 0 | 0 | 0 | 0 |
| IRS1 | 0.625 | 0 | 0 | 0 | 0 | 0.125 | 0 | 0 | 0 | 0 | 0 | 0.125 | 0 | 0 | 0 | 0 | 0 |
| JNK1 | 0.75 | 0 | 0 | 0 | 0 | 0.125 | 0 | 0 | 0 | 0 | 0 | 0.125 | 0.125 | 0.125 | 0 | 0 | 0.125 |
| MEK | 0.375 | 0 | 0 | 0 | 0 | 0.125 | 0 | 0 | 0 | 0 | 0 | 0.125 | 0 | 0 | 0 | 0 | 0 |
| MEKK1ASK1 | 0.5 | 0.125 | 0 | 0 | 0.125 | 0.125 | 0.125 | 0 | 0.125 | 0.125 | 0.125 | 0.125 | 0.125 | 0.125 | 0.125 | 0 | 0.125 |
| MK2 | 0.75 | 0 | 0 | 0 | 0 | 0.125 | 0 | 0 | 0 | 0.125 | 0 | 0.125 | 0 | 0 | 0.125 | 0 | 0 |
| MKK3 | 0.75 | 0 | 0 | 0 | 0 | 0.125 | 0 | 0 | 0 | 0.125 | 0 | 0.125 | 0 | 0 | 0.125 | 0 | 0 |
| MKK7 | 0.75 | 0 | 0 | 0 | 0 | 0.125 | 0 | 0 | 0 | 0 | 0 | 0.125 | 0.125 | 0.125 | 0 | 0 | 0.125 |
| NFκB | 0.75 | 0 | 0 | 0 | 0 | 0.125 | 0 | 0 | 0 | 0 | 0 | 0 | 0 | 0 | 0 | 0 | 0 |
| p38 | 0.75 | 0 | 0 | 0 | 0 | 0.125 | 0 | 0 | 0 | 0.125 | 0 | 0.125 | 0 | 0 | 0.125 | 0 | 0 |
