# Supplementary material for: Advanced Fault Diagnosis Methods in Molecular Networks
Source: PLoS One. 2014 Oct 7;9(10):e108830. doi: 10.1371/journal.pone.0108830 (PMC4188586; doi:10.1371/journal.pone.0108830)
Supplement: Table S3 — Sorted Network Vulnerabilities for All Pairs of Faulty Molecules in the Caspase3 Network. (DOCX) [file pone.0108830.s003.docx]

**Table S3:** Sorted Network Vulnerabilities for All Pairs of Faulty Molecules

| Faulty Pairs | | *V* |
| --- | --- | --- |
| AKT | cFLIPL | 0.75 |
| AKT | ComplexI | 0.75 |
| AKT | ComplexII | 0.75 |
| AKT | IKK | 0.75 |
| AKT | JNK1 | 0.75 |
| AKT | MK2 | 0.75 |
| AKT | MKK3 | 0.75 |
| AKT | MKK7 | 0.75 |
| AKT | NFκB | 0.75 |
| AKT | p38 | 0.75 |
| AKT | EGFR | 0.625 |
| AKT | IRS1 | 0.625 |
| AKT | MEKK1ASK1 | 0.5 |
| AKT | caspase8 | 0.375 |
| AKT | ERK | 0.375 |
| AKT | MEK | 0.375 |
| caspase8 | ComplexI | 0.125 |
| ComplexI | ComplexII | 0.125 |
| caspase8 | EGFR | 0.125 |
| cFLIPL | EGFR | 0.125 |
| ComplexI | EGFR | 0.125 |
| ComplexII | EGFR | 0.125 |
| EGFR | ERK | 0.125 |
| EGFR | IKK | 0.125 |
| EGFR | IRS1 | 0.125 |
| EGFR | JNK1 | 0.125 |
| EGFR | MEK | 0.125 |
| caspase8 | MEKK1ASK1 | 0.125 |
| ComplexII | MEKK1ASK1 | 0.125 |
| EGFR | MEKK1ASK1 | 0.125 |
| ERK | MEKK1ASK1 | 0.125 |
| IRS1 | MEKK1ASK1 | 0.125 |
| JNK1 | MEKK1ASK1 | 0.125 |
| MEK | MEKK1ASK1 | 0.125 |
| EGFR | MK2 | 0.125 |
| JNK1 | MK2 | 0.125 |
| MEKK1ASK1 | MK2 | 0.125 |
| EGFR | MKK3 | 0.125 |
| JNK1 | MKK3 | 0.125 |
| MEKK1ASK1 | MKK3 | 0.125 |
| EGFR | MKK7 | 0.125 |
| MEKK1ASK1 | MKK7 | 0.125 |
| MK2 | MKK7 | 0.125 |
| MKK3 | MKK7 | 0.125 |
| EGFR | NFκB | 0.125 |
| EGFR | p38 | 0.125 |
| JNK1 | p38 | 0.125 |
| MEKK1ASK1 | p38 | 0.125 |
| MKK7 | p38 | 0.125 |
| The rest of faulty pairs | | 0 |
