# Supplementary material for: Advanced Fault Diagnosis Methods in Molecular Networks
Source: PLoS One. 2014 Oct 7;9(10):e108830. doi: 10.1371/journal.pone.0108830 (PMC4188586; doi:10.1371/journal.pone.0108830)
Supplement: Table S4 — Average of All Double Fault Vulnerabilities Associated with each Molecule, when Simultaneously Faulty with Other Molecules in the Caspase3 Network. (DOCX) [file pone.0108830.s004.docx]

**Table S****4:** Average of All Double Fault Vulnerabilities Associated with each Molecule, when Simultaneously Faulty with Other Molecules

|  | AKT | caspase8 | cFLIPL | ComplexI | ComplexII | EGFR | ERK | IKK | IRS1 | JNK1 | MEK | MEKK1ASK1 | MK2 | MKK3 | MKK7 | NFκB | p38 |
| --- | --- | --- | --- | --- | --- | --- | --- | --- | --- | --- | --- | --- | --- | --- | --- | --- | --- |
| Average | 0.648 | 0.047 | 0.055 | 0.070 | 0.070 | 0.156 | 0.039 | 0.055 | 0.055 | 0.086 | 0.039 | 0.117 | 0.078 | 0.078 | 0.086 | 0.055 | 0.078 |
| Single | 0.75 | 0 | 0 | 0 | 0 | 0.125 | 0 | 0 | 0 | 0 | 0 | 0.125 | 0 | 0 | 0 | 0 | 0 |

The second row shows vulnerabilities for each single faulty molecule.
