# Supplementary material for: Advanced Fault Diagnosis Methods in Molecular Networks
Source: PLoS One. 2014 Oct 7;9(10):e108830. doi: 10.1371/journal.pone.0108830 (PMC4188586; doi:10.1371/journal.pone.0108830)
Supplement: Table S5 — Ternary Equations for the Caspase3 Network. (DOCX) [file pone.0108830.s005.docx]

**Table S****5:**  Ternary Equations for the Caspase3 Network in Figure 1

|  | Molecules | Ternary equations |
| --- | --- | --- |
| Molecules in the network (listed  alphabetically) | AKT | AKT= Max(EGFR,insulin) |
|  | caspase8 | caspase8= Min((1-cFLIPL) ,Max(ComplexII,ERK)) |
|  | cFLIPL | cFLIPL=NFκB |
|  | ComplexI | ComplexI=TNF |
|  | ComplexII | ComplexII= Max(TNF,ComplexI) |
|  | EGFR | EGFR=EGF |
|  | ERK | ERK=MEK |
|  | IKK | IKK=ComplexI |
|  | IRS1 | IRS1=Insulin |
|  | JNK1 | JNK1=MKK7 |
|  | MEK | MEK= Max(EGFR,IRS1) |
|  | MEKK1ASK1 | MEKK1ASK1=ComplexI |
|  | MK2 | MK2=p38 |
|  | MKK3 | MKK3=MEKK1ASK1 |
|  | MKK7 | MKK7=MEKK1ASK1 |
|  | NFκB | NFκB=IKK |
|  | p38 | p38=MKK3 |
| Network output molecule $\to$ | caspase3 | caspase3= Min((1-AKT),Max(caspase8,JNK1,MK2)) |

Each equation specifies the input signal(s) of a molecule in the network. The operations Max and Min represent maximum and minimum, respectively.
