# Supplementary material for: Advanced Fault Diagnosis Methods in Molecular Networks
Source: PLoS One. 2014 Oct 7;9(10):e108830. doi: 10.1371/journal.pone.0108830 (PMC4188586; doi:10.1371/journal.pone.0108830)
Supplement: Table S6 — Network Output for Some Single and Double Faulty Molecules in the Caspase3 Network (Incorrect Outputs Are Italic and Bold). (DOCX) [file pone.0108830.s006.docx]

**Table S****6:** Network Output for Some Single and Double Faulty Molecules (Incorrect Outputs Are Italic and Bold)

| EGF,insulin,TNF | 000 | 001 | 010 | 011 | 100 | 101 | 110 | 111 |
| --- | --- | --- | --- | --- | --- | --- | --- | --- |
| AKT=sa0 | 0 | 1 | ***1*** | ***1*** | ***1*** | ***1*** | ***1*** | ***1*** |
| p38=sa0 | 0 | 1 | 0 | 0 | 0 | 0 | 0 | 0 |
| MEKK1ASK1=sa0 | 0 | ***0*** | 0 | 0 | 0 | 0 | 0 | 0 |
| AKT=sa0 and p38=sa0 | 0 | 1 | ***1*** | ***1*** | ***1*** | ***1*** | ***1*** | ***1*** |
| AKT=sa0 and MEKK1ASK1=sa0 | 0 | ***0*** | ***1*** | 0 | ***1*** | 0 | ***1*** | 0 |
