# Supplementary material for: Advanced Fault Diagnosis Methods in Molecular Networks
Source: PLoS One. 2014 Oct 7;9(10):e108830. doi: 10.1371/journal.pone.0108830 (PMC4188586; doi:10.1371/journal.pone.0108830)
Supplement: Table S7 — Binary Equations for the SHP2 Network. (DOCX) [file pone.0108830.s007.docx]

**Table S****7:**  Binary equations for the SHP2 network in Figure 11A

| **Molecules** | **Binary equations** |
| --- | --- |
| abl(t) | abl(t)=lckp1(t)\|fyn(t) |
| cblb(t+1) | cblb(t+1)=~cd28 |
| ccblp1(t+1) | ccblp1(t+1)=zap70(t) |
| ccblp2(t+1) | ccblp2(t+1)=fyn(t) |
| cd28 | Input node |
| cd4 | Input node |
| cd45 | cd45=1 |
| csk(t) | csk(t)=pag(t) |
| dag(t) | dag(t)=(~dgk(t))&plcga(t) |
| dgk(t+1) | dgk(t+1)=tcrb(t) |
| erk(t) | erk(t)=mek(t) |
| fyn(t) | fyn(t)=tcrb(t)\|(lckp1(t)&cd45) |
| gab2(t+1) | gab2(t+1)=lat(t)&zap70(t)&(gads(t)\|grb2(t)) |
| gads(t) | gads(t)=lat(t) |
| gap | gap=0 |
| grb2(t) | grb2(t)=lat(t) |
| itk(t) | itk(t)=slp76(t)&zap70(t)&pip3(t) |
| lat(t) | lat(t)=zap70(t) |
| lckp1(t) | lckp1(t)=(~shp1(t))&(~csk(t))&cd45&cd4 |
| lckp2(t) | lckp2(t)=tcrb(t) |
| mek(t) | mek(t)=raf(t) |
| pag(t) | pag(t)=~tcrb(t) |
| pag(t+1) | pag(t+1)=fyn(t) |
| pi3k(t) | pi3k(t)=((~cblb(t))&x(t))\|((~cblb(t))&lckp2(t)) |
| pip3(t) | pip3(t)=pi3k(t)&(~ship1)&(~pten) |
| plcga(t) | plcga(t)=plcgb(t)&(~ccblp2(t))&slp76(t)&zap70(t)&vav1(t)&(itk(t)\|rlk(t)) |
| plcgb(t) | plcgb(t)=lat(t) |
| pten | pten=0 |
| raf(t) | raf(t)=ras(t) |
| ras(t) | ras(t)=(~gap)&rasgrp(t)&sos(t) |
| rasgrp(t) | rasgrp(t)=dag(t) |
| rlk(t) | rlk(t)=lckp1(t) |
| sh3bp2(t) | sh3bp2(t)=zap70(t)&lat(t) |
| ship1 | ship1=0 |
| shp1(t+1) | shp1(t+1)=(~erk(t))&lckp1(t) |
| shp2(t) | shp2(t)=gab2(t) |
| slp76(t) | slp76(t)=(~gab2(t))&zap70(t)&gads(t) |
| sos(t) | sos(t)=grb2(t) |
| tcrb(t) | tcrb(t)=(~ccblp1(t))&tcrlig |
| tcrlig | Input node |
| tcrp(t) | tcrp(t)=(tcrb(t)&lckp1(t))\|(tcrb(t)&fyn(t)) |
| vav1(t) | vav1(t)=(sh3bp2(t)&zap70(t))\|x(t) |
| x(t) | x(t)=cd28 |
| zap70(t) | zap70(t)=(~ccblp1(t))&abl(t)&tcrp(t) |

Each binary equation specifies the input signals to a molecule using the binary operations ~, | and &, which represent NOT, OR and AND, respectively. The symbol t represents the current time whereas t+1 symbolically stands for the next time interval.
