# Supplementary material for: Advanced Fault Diagnosis Methods in Molecular Networks
Source: PLoS One. 2014 Oct 7;9(10):e108830. doi: 10.1371/journal.pone.0108830 (PMC4188586; doi:10.1371/journal.pone.0108830)
Supplement: Methods S1 — (DOCX) [file pone.0108830.s008.docx]

# Supporting Information

# Methods S1

## Network matrices for all pairs of faulty molecules

Here network matrices for the caspase network are provided, when two molecules are simultaneously dysfunctional in the network. Each element of these matrices is a conditional probability of the form $P(\text{caspase3}|\text{EGF},\text{insulin},\text{TNF})$:

## Network matrices for the ternary model

Here network matrices for ternary model of the caspase network are provided. Equation is for the case when there is no faulty molecule in the network, whereas - represent the network when there is a faulty molecule. Each element of these matrices is a conditional probability of the form $P(\text{caspase3}|\text{EGF},\text{insulin},\text{TNF})$. For any given input pattern, this specifies the probability of the output to be 0, 1/2 or 1.

.

Equation is the network matrix **M** for the ternary model, when the faulty molecule is AKT.

$\begin{matrix} \text{AKT is faulty} \\ \text{with probability }p \end{matrix} \mathbf{M}=\left[\begin{matrix} 1 & 0 & 0 \\ 0 & 1 & 0 \\ 0 & 0 & 1 \\ 0 & 1 & 0 \\ 0 & 1 & 0 \\ 0 & 1-p & p \\ 1-p & 0 & p \\ 1-p & p & 0 \\ 1-p & 0 & p \\ 0 & 1 & 0 \\ 0 & 1 & 0 \\ 0 & 1-p & p \\ 0 & 1 & 0 \\ 0 & 1 & 0 \\ 0 & 1-p & p \\ 1-p & 0 & p \\ 1-p & p & 0 \\ 1-p & 0 & p \\ 1-p & 0 & p \\ 1-p & p & 0 \\ 1-p & 0 & p \\ 1-p & 0 & p \\ 1-p & p & 0 \\ 1-p & 0 & p \\ 1-p & 0 & p \\ 1-p & p & 0 \\ 1-p & 0 & p \end{matrix}\right]$.

Equation is the network matrix **M** for the ternary model, when the faulty molecule is EGFR.

$$\begin{matrix} \text{EGFR is faulty} \\ \text{ with probability }p \end{matrix} \mathbf{M}=\left[\begin{matrix} 1 & 0 & 0 \\ 0 & 1 & 0 \\ 0 & 0 & 1 \\ 0 & 1 & 0 \\ 0 & 1 & 0 \\ 0 & 1 & 0 \\ 1 & 0 & 0 \\ 1 & 0 & 0 \\ 1 & 0 & 0 \\ p & 1-p & 0 \\ 0 & 1 & 0 \\ 0 & 1-p & p \\ 0 & 1 & 0 \\ 0 & 1 & 0 \\ 0 & 1 & 0 \\ 1 & 0 & 0 \\ 1 & 0 & 0 \\ 1 & 0 & 0 \\ 1 & 0 & 0 \\ 1-p & p & 0 \\ 1-p & 0 & p \\ 1-p & p & 0 \\ 1-p & p & 0 \\ 1-p & p & 0 \\ 1 & 0 & 0 \\ 1 & 0 & 0 \\ 1 & 0 & 0 \end{matrix}\right]$$

Equation is the network matrix **M** for the ternary model, when the faulty molecule is MEKK1ASK1.

$$\begin{matrix} \text{MEKK1ASK1} \\ \text{is faulty} \text{with} \\ \text{probability }p \end{matrix} \mathbf{M}=\left[\begin{matrix} 1 & 0 & 0 \\ 0 & 1 & 0 \\ p & 0 & 1-p \\ 0 & 1 & 0 \\ 0 & 1 & 0 \\ p & 1-p & 0 \\ 1 & 0 & 0 \\ 1 & 0 & 0 \\ 1 & 0 & 0 \\ 0 & 1 & 0 \\ 0 & 1 & 0 \\ p & 1-p & 0 \\ 0 & 1 & 0 \\ 0 & 1 & 0 \\ p & 1-p & 0 \\ 1 & 0 & 0 \\ 1 & 0 & 0 \\ 1 & 0 & 0 \\ 1 & 0 & 0 \\ 1 & 0 & 0 \\ 1 & 0 & 0 \\ 1 & 0 & 0 \\ 1 & 0 & 0 \\ 1 & 0 & 0 \\ 1 & 0 & 0 \\ 1 & 0 & 0 \\ 1 & 0 & 0 \end{matrix}\right]$$

Equation is the network matrix **M** for the ternary model, when the faulty molecule is caspase8, ERK or MEK.

$$\begin{matrix} \text{Caspase8},\text{ERK or MEK} \\ \text{is faulty} \text{with} \\ \text{probability }p \end{matrix} \mathbf{M}=\left[\begin{matrix} 1 & 0 & 0 \\ 0 & 1 & 0 \\ 0 & 0 & 1 \\ p & 1-p & 0 \\ 0 & 1 & 0 \\ 0 & 1 & 0 \\ 1 & 0 & 0 \\ 1 & 0 & 0 \\ 1 & 0 & 0 \\ p & 1-p & 0 \\ 0 & 1 & 0 \\ 0 & 1 & 0 \\ p & 1-p & 0 \\ 0 & 1 & 0 \\ 0 & 1 & 0 \\ 1 & 0 & 0 \\ 1 & 0 & 0 \\ 1 & 0 & 0 \\ 1 & 0 & 0 \\ 1 & 0 & 0 \\ 1 & 0 & 0 \\ 1 & 0 & 0 \\ 1 & 0 & 0 \\ 1 & 0 & 0 \\ 1 & 0 & 0 \\ 1 & 0 & 0 \\ 1 & 0 & 0 \end{matrix}\right]$$

Equation is the network matrix **M** for the ternary model, when the faulty molecule is IRS1.

$$\begin{matrix} \text{IRS1} \\ \text{is faulty} \text{with} \\ \text{probability }p \end{matrix} \mathbf{M}=\left[\begin{matrix} 1 & 0 & 0 \\ 0 & 1 & 0 \\ 0 & 0 & 1 \\ p & 1-p & 0 \\ 0 & 1 & 0 \\ 0 & 1 & 0 \\ 1 & 0 & 0 \\ 1 & 0 & 0 \\ 1 & 0 & 0 \\ 0 & 1 & 0 \\ 0 & 1 & 0 \\ 0 & 1 & 0 \\ 0 & 1 & 0 \\ 0 & 1 & 0 \\ 0 & 1 & 0 \\ 1 & 0 & 0 \\ 1 & 0 & 0 \\ 1 & 0 & 0 \\ 1 & 0 & 0 \\ 1 & 0 & 0 \\ 1 & 0 & 0 \\ 1 & 0 & 0 \\ 1 & 0 & 0 \\ 1 & 0 & 0 \\ 1 & 0 & 0 \\ 1 & 0 & 0 \\ 1 & 0 & 0 \end{matrix}\right]$$

Equation is the network matrix **M** for the ternary model, when the faulty molecule is IKK, ComplexI, ComplexII, NFκB, JNK1, MK2, cFLIPL, MKK3, MKK7 or p38.

$$\begin{matrix} \text{ IKK, ComplexI,} \\ \text{ComplexII}, \text{NF}\kappa\text{B,} \\ \text{JNK1}, \text{MK2},\text{cFLIPL}, \\ \text{MKK3}, \text{MKK7},\text{or} \text{p38} \\ \text{is} \text{faulty} \text{with} \\ \text{probability }p \end{matrix} \mathbf{M}=\left[\begin{matrix} 1 & 0 & 0 \\ 0 & 1 & 0 \\ 0 & 0 & 1 \\ 0 & 1 & 0 \\ 0 & 1 & 0 \\ 0 & 1 & 0 \\ 1 & 0 & 0 \\ 1 & 0 & 0 \\ 1 & 0 & 0 \\ 0 & 1 & 0 \\ 0 & 1 & 0 \\ 0 & 1 & 0 \\ 0 & 1 & 0 \\ 0 & 1 & 0 \\ 0 & 1 & 0 \\ 1 & 0 & 0 \\ 1 & 0 & 0 \\ 1 & 0 & 0 \\ 1 & 0 & 0 \\ 1 & 0 & 0 \\ 1 & 0 & 0 \\ 1 & 0 & 0 \\ 1 & 0 & 0 \\ 1 & 0 & 0 \\ 1 & 0 & 0 \\ 1 & 0 & 0 \\ 1 & 0 & 0 \end{matrix}\right]$$

## Equations for vulnerabilities in terms of the input activities

The vulnerability formula in (7) is for the case when inputs are equi-probable. When inputs have different activity levels, we can compute the vulnerability of a network to the dysfunction of each individual molecule, by including input activities $q_{1},q_{2} \text{and} q_{3}$ in (6), as follows:

$$\begin{matrix} V=P(0|001)(1-q_{1})(1-q_{2})q_{3}+P(1|000)(1-q_{1})(1-q_{2})(1-q_{3}) +P(1|010)(1-q_{1})q_{2}(1-q_{3})+P(1|011)(1-q_{1})q_{2}q_{3} \\ +P(1|100)q_{1}(1-q_{2})(1-q_{3})+P(1|101)q_{1}(1-q_{2})q_{3}+P(1|110)q_{1}q_{2}(1-q_{3})+P(1|111)q_{1}q_{2}q_{3}. \end{matrix}$$

By replacing the above conditional probabilities with the elements of the matrices in (2)-(5), the following equations can be obtained for the vulnerability of each molecule in terms of its fault probability *p* and the input activities $q_{1},q_{2} \text{and} q_{3}$:

$$V=\left\{\begin{matrix} p(q_{1}+q_{2}-q_{1}q_{2}), & \text{AKT} \text{is faulty,} \\ pq_{1}(1-q_{2})q_{3}, & \text{EGFR} \text{is faulty,} \\ p(1-q_{1})(1-q_{2})q_{3}, & \text{MEKK1ASK1} \text{is faulty,} \\ 0, & \text{otherwise.} \end{matrix}\right.$$

For $q_{1}=q_{2}=q_{3}=1/2$, reduces to (8).
